# Large scale systematic proteomic quantification from non-metastatic to metastatic colorectal cancer

**DOI:** 10.1038/srep12120

**Published:** 2015-07-15

**Authors:** Xuefei Yin, Yang Zhang, Shaowen Guo, Hong Jin, Wenhai Wang, Pengyuan Yang

**Affiliations:** 1 Department of Systems Biology for Medicine, Fudan University Shanghai Medical College, Shanaghai 200032, China; 2 Department of Chemistry and Institutes of Biomedical Sciences of Shanghai medical School, Fudan University, Shanghai 200032, China; 3 Shuguang Hospital affiliated to Shanghai University of Traditional Chinese Medicine, Shanghai 201203, China

## Abstract

A systematic proteomic quantification of formalin-fixed, paraffin-embedded (FFPE) colorectal cancer tissues from stage I to stage IIIC was performed in large scale. 1017 proteins were identified with 338 proteins in quantitative changes by label free method, while 341 proteins were quantified with significant expression changes among 6294 proteins by iTRAQ method. We found that proteins related to migration expression increased and those for binding and adherent decreased during the colorectal cancer development according to the gene ontology (GO) annotation and ingenuity pathway analysis (IPA). The integrin alpha 5 (ITA5) in integrin family was focused, which was consistent with the metastasis related pathway. The expression level of ITA5 decreased in metastasis tissues and the result has been further verified by Western blotting. Another two cell migration related proteins vitronectin (VTN) and actin-related protein (ARP3) were also proved to be up-regulated by both mass spectrometry (MS) based quantification results and Western blotting. Up to now, our result shows one of the largest dataset in colorectal cancer proteomics research. Our strategy reveals a disease driven omics-pattern for the metastasis colorectal cancer.

Over the past decades, colorectal cancer (CRC) become one of the most serious malignancies worldwide. CRC is the third most common cause of death among cancer patients in US^1^. Even though the development of modern systemic therapies for CRC, over 50% of patients will progress and develop metastases, whose prognosis is extremely poor and survival is limited once metastases become clinically evident^2^. In 2014, an estimated 71,830 men and 65,000 women will be diagnosed with colorectal cancer and 26,270 men and 24,040 women will die of the disease^1^. Therefore, the research on the mechanism of CRC development is very important, especially for the metastasis process. In recent years, it has gained considerable attention^3^ to investigate the difference CRC among normal mucosa, adenomas and cancer cells in CRC by using proteomic methods. Mass spectrometry based proteomics has increased the identified number of proteins from several hundred to thousands in combination of various separation approaches^4^,^5^,^6^. Especially the development of liquid chromatography coupled with tandem mass spectrometry (LC-MS/MS) technology in high resolution platforms^7^,^8^, both qualitative and quantitative analysis of low abundance proteins have been improved rapidly. MS based relative protein quantitative strategies can be mainly divided into stable isotope labeling (e.g. ^18^O, iTRAQ and SILAC)^9^ and label free (e.g. APEX, emPAI, iBAQ, Top3 and MeanInt)^10^ approaches. Quantitative proteomics is important for explaining the biological processes. Such advancements in proteomics opened up possibilities to screen out low abundance proteins as potential biomarkers in large scale^11^,^12^, which were not easily found by conventional approaches. Therefore, the simulation of biological processes according to different stages of cancer is available for system biology^13^. There are several of studies using quantitative proteomics approaches in which comparisons of the protein expression level changes between normal tissues and cancer tissue or cell lines have been made^14^,^15^,^16^ and the eventually related biomarkers for clinical diagnoses have been found by these studies. Unfortunately, there is no report for the difference of protein expression in the development of colorectal cancer.

Fresh tissues are very difficult to be collected in clinical proteomics study because in a clinical diagnosis the FFPE tissues are the most frequently used and easily to be preserved. However, it is difficult to recover intact protein from FFPE tissues for formalin-fixed,paraffin-embedded tissues had crosslinking effect^17^. Therefore, protein extraction from FFPE tissues is the essential step in proteomics sample preparation. In 2006, Shi *et al.*^18^ first published the work using gel electrophoresis followed by mass spectrometry to identify protein extracted from FFPE tissues. That indicated the potential use of the FFPE tissues in proteomic studies for most of the archived pathological specimens which were kept as FFPE tissues in hospitals for long-term preservation^19^. In the following, several groups have reported the application of the MS-based methods to analyze the proteome of FFPE tissues^20^,^21^,^22^.

Herein, we applied both label-free and iTRAQ based proteomic method to screen protein expression changes in five stages of colorectal cancer FFPE tissues for the first time. Finally, we reported relative quantification information of 1017 proteins by label-free and 6294 proteins by iTRAQ. And three potential metastasis biomarkers (ITA5, ARP3, VTN) were evaluated by functional annotation and validated by Western blotting.

## Results

### Experimental Design

**Figure 1:**
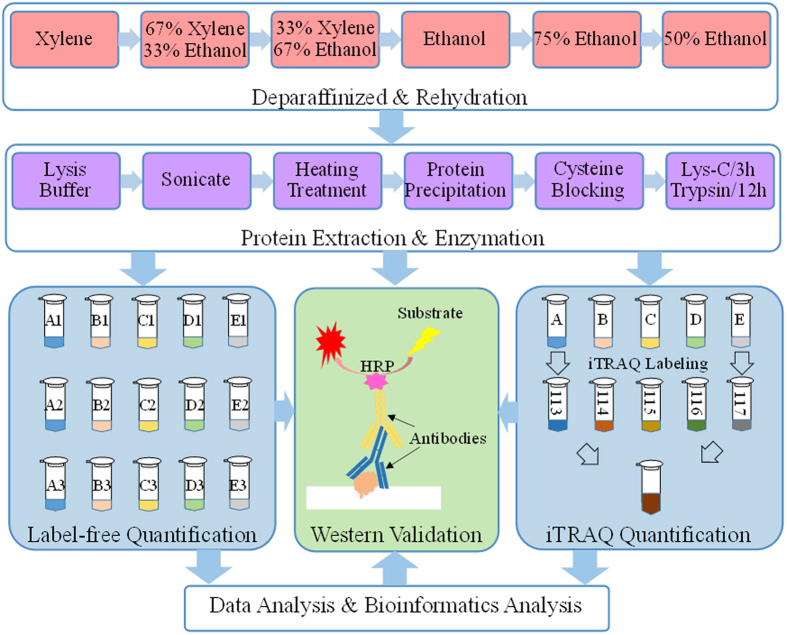
Quantitative proteomic workflow applied to colorectal cancer samples. Sample A represents Stages IIIC, Sample B represents Stages I, Sample C represents Stages II, Sample D represents Stages IIIB, Sample E represents Stages IIIA, of the clinical FFPE tissues respectively.

In order to evaluate differentially expressed proteins in the development of colorectal cancer, protein extracts from the 30 FFPE tissues representing five different clinical stages were used. As the workflow was shown in Fig. 1, the label-free and iTRAQ quantitative proteomic methods were applied to analyze the protein expression level of the five clinical CRC stages. According to bioinformatics analysis, the potential protein biomarkers were validated by Western Blotting.

### Label-free Quantification of FFPE Colorectal Tissues

**Figure 2:**
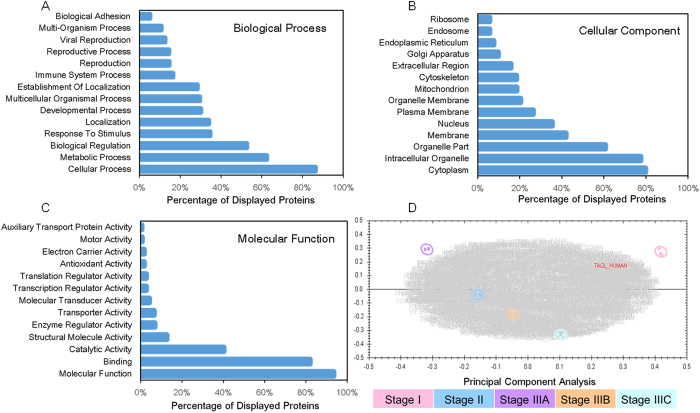
GO Annotation and Principal Component Analysis (PCA) of all identified protein from label free experiments. Biological processes (**A**) cellular component (**B**) and molecular function (**C**) overview of the colorectal cancer proteins using GO. The PCA of proteome dynamics based on protein abundance and turnover information generated by high-resolution mass spectrometry.

Protein quantification was carried out in the five category samples which were injected as triplicated, using MS1 peak intensity in Progenesis LC-MS. The 15 runs show a highly reproducibility for an average of 90% features were aligned. 20044 features were detected by software that exhibit at least 3 isotopic peaks with a charge state of [M+2H]^2+^, [M+3H]^3+^, or [M+4H]^4+^ in the compiled dataset. The files of peak list were corresponding to 1464 proteins and 6594 peptide identifications that were accepted at an FDR less than 1.0%. GO enrichment analysis was conducted on all identified proteins. An enrichment analysis of biological process annotations was shown in Fig. 2A–C described the localization as well as molecular functions with all the involved proteins. Then protein abundance was calculated from the sum of all ion abundances of normalized unique peptide for a specific protein on each run. Among the identified proteins, 1017 proteins had quantification information (Table S1). The results of PCA analysis by Progenesis LC-MS were visualized by the biplot (Fig. 2D). Transforming and plotting the abundance data in principal component space allowed us to separate the running samples according to protein expression variation. It will be helpful in interpreting relationships between the experimental groups. From the biplot, we can see that runs from the same group (the same colored dots) were close to each other, indicating that the results was highly reproducible among three technical replications and there were parts of protein abundance significantly different between groups. Based on ANOVA scores (p < 0.05) and max fold change >1.5, 338 proteins were finally found that their expression level were significantly changed during the colorectal cancer development from Stage I to Stage IIIC. Among these proteins, 75 proteins were down-regulated in metastasis cancer tissues.

The level of proteins expression changes were classified according to their biological functions and relationship to disease by using Ingenuity Pathway Analysis software. The top-ranked of up-regulation canonical pathways showed great relationship with energy metabolism, such as mitochondrial dysfunction, oxidative phosphorylation and noradrenaline and adrenaline degradation. The down-regulated proteins were related with canonical pathways, including ILK signaling, agranulocyte adhesion and diapedesis, and caveolar-mediated endocytosis signaling.

### iTRAQ Analysis of FFPE Colorectal Tissues

**Figure 3:**
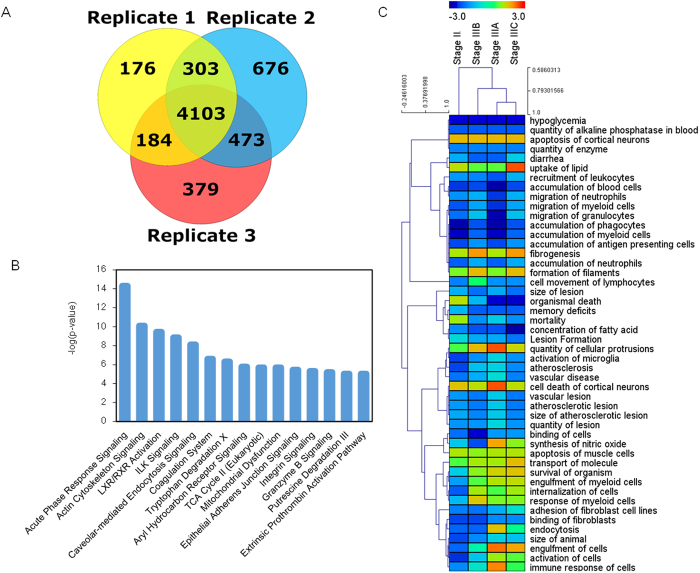
iTRAQ Analysis of FFPE Colorectal Tissues. (**A**) Venn diagram for the distribution of proteins quantification among the three iTRAQ experiments. (**B**) 15 most related canonical pathways in IPA. (**C**) Cluster analysis of the diseases and bio functions are summarized in a heat-map form.

To obtain the quantitative proteomics profiles in a larger scale and validation of our results in the previous, five category samples were analyzed using iTRAQ. Three technical replicates for the samples were compared to measure the variation. In those three analyses, 4766 (Replicate 1), 5555 (Replicate 2) and 5139 (Replicate 3) unique proteins were identified by iTRAQ method at an FDR lower than 1% (Fig. 3A), respectively. Then three replicated resulted of iTRAQ experiments were combined into a single one by using 1% FDR criteria at protein level. Combining the results of three replicates of iTRAQ experiments, 6294 unique proteins were finally identified with quantification information. Among them, 4103 were detected in each of the three iTRAQ experiments, and 960 were common in at least two runs. Approximately two-thirds of the proteins can be detected and quantified in all three replicate that indicated a good sample preparation technology and a good analytical reproducibility.

P-value was used to evaluate the significance of protein expression level change among the different stages of colorectal cancer tissues in iTRAQ. Stage I CRC was taken as a reference, the quantified protein was selected with p-value less than 0.05 which means the protein expression level significantly different from Stage I. By using this criteria, 105 proteins in Stage II, 186 proteins in Stage IIIA, 134 proteins in Stage IIIB and 115 proteins on Stage IIIC were observed significantly down-regulated or up-regulated, respectively, for each stage. These 341 proteins were all summarized in a quantified expression differential proteins list (Table S2). Among the differential expressed proteins, there are 140 proteins are consistence with laben-free quantification. In each Stage, up- and down- regulated proteins and their related processes and functions have been mining. Using the ingenuity pathway analysis tool, the canonical pathways at different stages were analyzed (Table S3) and the top 15 pathways were shown in Fig. 3B. We found that the canonical pathway related to the adherent and movement, such as actin cytoskeleton signaling, tight junction, epithelial adherence junction signaling and integrin signaling. The pathways are related to inflammation and immunity, such as acute phase response signaling, caveolar-mediated endocytosis signaling and agranulocyte adhesion and diapedesis. The significant expression level changes on these pathways are leading to the bio-functional changes and disease development. We illustrated all these changes in Table S4. From the dataset, on one hand, the proteins related to accumulation of phagocytes, blood cells, myeloid cells and antigen presenting cells, the cell death and apoptosis were down-regulated. On the other hands, the phagocytosis of cells and migration of cells were up-regulated, which indicating the increase of metastasis ability. Hierarchical clustering was analyzing of the five category samples using diseases and functions in IPA showing the correlation efficiency was higher between Stage IIIA-C. Top 50 significant changed diseases and bio functions were shown in Fig. 3C.

### Western Blotting Validation

**Figure 4:**
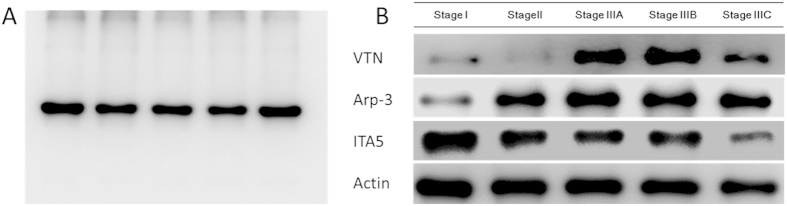
Western blotting Validation of proteomics results from different FFPE tissues. (**A**) Western-blotting is compatible with FFPE tissue extractions; (**B**) Western-blotting studies differential expression pattern of 3 candidate proteins: Up-regulation of VTN and ARP3 and down-regulation of ITA5 in metastasis tissues were observed.

There are several previous studies report the suitability of FFPE tissue extractions for Western blotting^23^,^24^. To validate the protein extractions used in this study, we performed the Western blotting by using beta-actin antibody. Although the tissues were not fresh, the western blotting showed that the bands corresponding to the beta-actin was distinct from the background (Fig. 4A). This experiment indicated that Western blotting is a compatible method with the extracted protein from FFPE tissues. Parallel western blotting of actin was used as a protein loading quantification control. The result of ITA5 overexpression in primary colorectal cancer was consistent with expected. ARP3, and VTN were observed to be overexpressed in clinical metastasis colorectal cancer tissues (Fig. 4B).

## Discussion

As the sample were chosen from Stage I to Stage IIIC, correspondent to cancer development from primary tumor to metastasis cancer. A considerable number of proteins involved in energy metabolism processes were up-regulated in our quantitative proteomics dataset for accelerated growth is a common feature of cancer cells and is reflected an increasing in the need of energy involved in processing^25^,^26^. This proved that cancer cells might need more energy during the development of cancer from in site tumor to metastasis cancer. As the data also shown, immune cell related migration and inflammatory responses were deceased, it supported that the colorectal cancer cell might escape the immune cell monitor during the cancer development^27^. Therefore, the colorectal cancer could be promoted from Stage I to Stage IIIC, which indicating potential pathways of the tumor cells using to escape from the immune system. On the other hands, the immune response of cells was upreglated in Stage IIIA-C, which indicate the two-sides roles of the immune system during colorectal cancer development^28^. Therefore, analyzing these samples give us information about the cause of tumor metastasis process. In the following, we focused our analysis on two bio-function relevant to metastasis: binding ability of cells and migration ability of cells. The integrin family was found playing an important role which influenced the migration ability. According to pathway analysis, the integrin signaling is one of the most significant down-regulated pathway from Stage I colorectal cancer to Stage III. Integrin proteins are a family of 24 different cell surface receptors which are comprised of non-covalently associated 8 β subunits and 18 α subunits^29^,^30^. 20 subunits of integrin were found in our data (Table 1).

**Figure 5:**
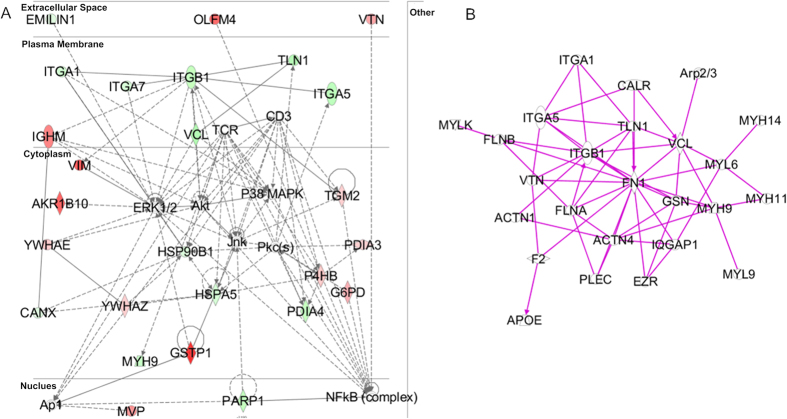
The enriched pathway of Integrin (**A**) and migration (**B**).

Integrin proteins are generally located on the cell surface which is involved in cell-cell and cell-extracellular matrix interactions. Integrin proteins have function to organize the cytoskeleton, activate intracellular signaling pathways, and also are important in cancer related pathways^31^,^32^, such as mitogen activated protein kinase (MAPK) pathways, Jun amino-terminal kinase (JNK) pathway and Extracellular signal regulated kinase (ERK) pathways(Fig. 5A). Among the identified 20 kinds of integrin proteins, ITA5, ITB1, ITA7 and ITA1 showed significant decrease expression and ITAL showed significant increase expression, that indicating the cancer development the cancer metastasis has a relationship with these proteins. And they might be potential biomarker of metastasis cancer. As the migration of cancer cells activated and binding or adherent inactivated, we combine the related molecules (Fig. 5B) from our pathway analysis in IPA. It shows that many proteins in integrin family are involved and many proteins related to actin reconstruction as well. Actin nucleation is the most important step of organize the cytoskeleton which can influence the cell movement. The Arp2/3 complex plays a central role in the pathway of actin nucleation by forming ARP-WASP complex, the ARP2/3 subunits is up-regulated indicating the increase of moving ability.

In order to further verify the data and analysis results finding by the label-free and iTRAQ proteomics, the selected candidate proteins were confirmed by Western blotting using commercially available antibodies. ITA5 was chosen from the integrin family for it was the only up regulated with 2 fold change in both quantitative methods. In addition, ARP3 and VTN were chosen because they were both in significant level change in the previous analysis. Although the results of Western blotting were not the same as MS-based quantification results in Table 2, the difference between non-metastasis(Stage I) and metastasis colorectal cancer(Stage IIIA-C) was fully consistent with iTRAQ and label free quantification. And from the data we can find that iTRAQ quantification is more consistent with the result in Western blotting for three out of four proteins.

In summary, our study provided a large scale systematic quantification for the differential expressed proteins during the cancer development from an early stage to stage IIIC. Approximately two-thirds of the proteins can be detected and quantified in all runs with relative lower false positive rates. Different quantification methods including label free quantification and iTRAQ were performed which corresponding to 1017 and 6294 proteins quantified, respectively. By using bioinformatics tools analysis, a disease driven omics-pattern for the potential mechanism of metastasis colorectal cancer were shown. A complete bioinformatics analysis using GO annotation and IPA were used to carry on data mining analysis. The proteins in integrin family and cell movement related proteins were focused to analyze the mechanism for metastasis cancer related pathways. Finally, the expression level of ARP3 and VTN increased while ITA5 decreased in metastasis tissues which were confirmed by western blotting and MS based quantification.

## Methods

### Chemicals and Materials

Water and ACN with 0.1% FA (LC-MS Ultra CHROMASOLV®, tested for UHPLC-MS), urea, sodium dodecyl sulfonate (SDS), ammonium bicarbonate, and tris base were purchased from Sigma Aldrich (USA). TCEP, MMTS, and TEAB were purchased from AB Sciex (USA). Cocktail of protease inhibitors, water and ACN were ordered from Thermo Fisher (USA). Trypsin (sequencing grade-modified) was obtained from Promega (USA). Mass spectrometry grade lysyl endoprotease was purchased from Wako (Japan).

### Sample Collection

All of the tissue specimens were obtained from the surgical resection in Shuguang Hospital affiliated to Shanghai University of Traditional Chinese medicine. The experiments were carried out in accordance with the approval notice() from IRB of Shuguang Hospital affiliated to Shanghai University of Traditional Chinese medicine. Tissues were collected after “informed consent” was given from the patients. Specific FFPE colorectal cancer tissue sections (~5 um thick) were collected in an Eppendorf tube. 30 tissue specimens are divided into five groups (A–E), corresponding to Stage I to Stage IIIC (Supplemental Table S5). All the candidates are under the standard TNM staging system clinical diagnosis condition.

### Preparation of Cell Lysates

Tissue sections were deparaffinized by incubating in a graded series of xylene (100%, 67% and 33%) for 2 min each at room temperature. Then, the tissue sections were rehydration in a graded of ethanol (100%, 75% and 50%). The lysis buffers(4% SDS, 0.1 M Tris-HCl, pH 8.0) were added in the tube, followed incubating at 99 °C for 30 min, and at 60 °C for 2 h with shaking. The liquid tissue extracts were centrifuged for 10 min at 14000 g at 4 °C. The extracted protein was mixed according to groups (A–E) in Table S5 with equal amount and then precipitated with acetone overnight. After re-suspending the protein in TEAB buffer, protein quantification was done using the BCA Kit (Thermo Fisher Scientific).

### In-solution digestion/High pH RPLC

The proteins were reduced by 5 mM DTT at 56 °C for 30 min and alkylated by 10 mM MMTS at room temperature for 30 min. And then the sample was diluted with 50 mM ammonium bicarbonate until the concentration of urea was lower than 1 M. Lys-C was added into proteins at the mass ratio of 1:50 (enzyme : protein) for 3 hours at 37 °C. Then, and trypsin was added to the sample at the mass ratio of 1:50 (enzyme: protein) for 12 hours. For label-free quantification, the digested peptides were desalted using a C18 column (Sep-Pak Vac C18, Waters Corporation), concentrated using a SpeedVac, and then resuspended in 2% ACN with 0.1% FA. For iTRAQ samples, iTRAQ-8plex labeling reagents (AB Sciex) were added to the peptide samples, which were incubated at room temperature for 120 min. The reaction was stopped by the addition of water, followed concentration using SpeedVac and desalts. The digested protein samples were fractionated by using high pH reversed phase liquid chromatography.

### UHPLC-MS/MS

Nano-UHPLC was performed on Eksigent 1D plus with 15 cm column from Eksigent (C18 3 μm, 120 Å, 75 μm × 15 cm). Digested protein samples were automatically loaded onto the trap column from Eksigent (C18 3 μm, 350 μm × 0.5 mm, 120 Å). For gradient elution analysis, the mobile phases consisted of (A) 0.1% FA in 2% ACN and (B) 0.1% FA in consisted of 98% ACN. Peptides were eluted from 15 cm column at the constant flow rate of 300 nL/min in a 90 min gradient condition. Triple TOF4600 and Triple TOF5600+ (AB Sciex) were operated in positive mode with an ion spray voltage at 2.3 kV. Survey scans were acquired from 350 to 1500 m/z in high resolution mode while MS/MS scans were from 100 to 1250 m/z in high sensitivity mode. 20–40 most intensive precursors were selected for fragmentation per cycle separately.

### SDS-PAGE and Western Blotting

The protein sample was mixed with SDS-PAGE loading buffer and heated at 100 °C for 5 min before separation on a gel. Total 20 μg of extracted protein was separated with a 12% SDS-PAGE gel and the standard protein ladder was used as references of molecular weights (MWs). The proteins were transferred to polyvinylidene fluoride (PVDF) membranes using Bio-Rad’s western blotting system at 80 V for 2 h without staining. The membranes were blocked using 5% skimmed milk in TBS for 2 h at room temperature, washed three times in TBST for 5 min, and incubated over night at 4 °C with monoclonal antibodies against actin(1:5000), ITGA5(1:500) from Immunoway, ARP3(1:1000) from Epitomics, Vitronectin(1:1000) from abcam. After washing three times in TBST for 5 min, the secondary antibody was added and incubated for 1 h at room temperature. Finally, the ECL system (GE Healthcare) was used after 5 min washing in TBST three times for the membrane.

### Database Searches and Quantitative Proteome Analysis

All tandem mass spectra of label-free samples were extracted by Progenesis LC-MS, and then were analyzed by Mascot (Matrix Science, London, UK; version 2.4.1), which was set up to search against the Swiss Prot database (selected for Homo sapiens, 20267 entries) with trypsin as digestion enzyme. The Mascot was searched with a fragment ion mass tolerance of 0.1 Da and a parent ion tolerance of 25 ppm. MMTS modification of cysteine residues was specified in Mascot as a fixed modification. Oxidation of methionine and acetyl of the protein N-terminus were set in Mascot as variable modifications. Scaffold (version Scaffold_4.2.1, Proteome Software Inc., Portland, OR) was used to validate for MS/MS based peptide and protein identifications. Peptide identifications were accepted if their FDR value was less than 1.0%, while protein identifications had the same and contained at least 2 identified peptides. For iTRAQ experiments, protein identification and iTRAQ 8 plex quantification were performed with ProteinPilot4.5 software. A decoy database search strategy was adopted to estimate the FDR <1% for peptide and protein identification.

## Additional Information

**How to cite this article**: Yin, X. *et al.* Large scale systematic proteomic quantification from non-metastatic to metastatic colorectal cancer. *Sci. Rep.* **5**, 12120; doi: 10.1038/srep12120 (2015).

## Supplementary Material

Supplementary Table S1

Supplementary Table S2

Supplementary Table S3

Supplementary Table S4

Supplementary Table S5

## Figures and Tables

**Table 1:** List of integrin family proteins quantified by iTRAQ experiments^a^.

			iTRAQ Values			
Accession	Name	Unused	Stage II/Stage I	Stage IIIA/Stage I	Stage IIIB/Stage I	Stage IIIC/Stage I
ITB3_HUMAN	Integrin beta-3	3.6	1.08	0.89	1.06	0.85
ITB2_HUMAN	Integrin beta-2	23.1	0.91	0.84	1.16	0.92
ITB1_HUMAN	Integrin beta-1	27.8	0.22	0.48	0.48	0.66
ITAV_HUMAN	Integrin alpha-V	34.7	0.71	0.82	1.12	0.88
ITA2B_HUMAN	Integrin alpha-IIb	7.5	0.95	0.67	0.93	0.69
ITA5_HUMAN	Integrin alpha-5	26.7	0.32	0.37	0.41	0.38
ITAM_HUMAN	Integrin alpha-M	35.7	0.74	0.81	1.35	0.67
ITB4_HUMAN	Integrin beta-4	24.9	0.77	1.20	0.76	0.88
ITA2_HUMAN	Integrin alpha-2	16.9	1.01	1.66	1.30	1.40
ITB5_HUMAN	Integrin beta-5	4.8	0.86	0.81	1.16	0.94
ITAL_HUMAN	Integrin alpha-L	5.6	0.84	2.17	2.48	1.59
ITAX_HUMAN	Integrin alpha-X	4.5	0.90	0.83	1.04	0.89
ITA6_HUMAN	Integrin alpha-6	35.8	0.77	1.10	0.94	0.77
ITA6_HUMAN	Integrin alpha-6	38.9	0.53	1.47	1.24	0.82
ITA3_HUMAN	Integrin alpha-3	10.2	0.89	0.98	1.03	1.00
ITB7_HUMAN	Integrin beta-7	4.0	1.63	1.84	1.36	1.53
ITAE_HUMAN	Integrin alpha-E	0.5	0.10	14.32	2.94	14.06
ITA1_HUMAN	Integrin alpha-1	21.1	0.37	0.54	0.60	0.87
ITA7_HUMAN	Integrin alpha-7	16.0	0.56	0.33	0.64	0.65
ITA11_HUMAN	Integrin alpha-11	2.2	0.88	0.84	0.96	0.77

^a^The list contains information about the integrin family in average from the three biological replicates of the iTRAQ experiment.

**Table 2:** The Protein abundance quantified by iTRAQ and Label-free^a^.

Quantification Methods	Accession	Name	Stage I	Stage II	Stage IIIA	Stage IIIB	Stage IIIC
iTRAQ	VTNC_HUMAN	Vitronectin	control	3.20	0.77	2.19	4.56
	ITA5_HUMAN	Integrin alpha-5	control	0.32	0.37	0.41	0.38
	ARP3_HUMAN	Actin-related protein 3	control	1.59	1.63	1.92	1.85
	ACTB_HUMAN	Actin, cytoplasmic 1	control	0.95	1.26	1.17	0.95
Label Free	VTNC_HUMAN	Vitronectin	control	2.10	0.87	1.37	2.70
	ITA5_HUMAN	Integrin alpha-5	control	0.53	0.44	0.47	0.41
	ARP3_HUMAN	Actin-related protein 3	control	0.83	0.87	0.99	1.00
	ACTB_HUMAN	Actin, cytoplasmic 1	control	1.04	1.02	1.05	1.01

^a^The iTRAQ Value and Average Normalised Abundances were used to representative the abundance of protein.
